# Novel and Recurrent Copy Number Variants in *ABCA4*-Associated Retinopathy

**DOI:** 10.3390/ijms25115940

**Published:** 2024-05-29

**Authors:** Zelia Corradi, Claire-Marie Dhaenens, Olivier Grunewald, Ipek Selen Kocabaş, Isabelle Meunier, Sandro Banfi, Marianthi Karali, Frans P. M. Cremers, Rebekkah J. Hitti-Malin

**Affiliations:** 1 Department of Human Genetics, Radboud University Medical Center, 6525 GA Nijmegen, The Netherlands; 2 Université de Lille, Inserm, CHU Lille, U1172-LilNCog-Lille Neuroscience & Cognition, F-59000 Lille, France; 3 Institute des Neurosciences de Montpellier, INSERM, Université de Montpellier, F-34295 Montpellier, France; 4 Department of Precision Medicine, University of Campania “Luigi Vanvitelli”, 81031 Naples, Italy; 5 Telethon Institute of Genetics and Medicine (TIGEM), 80078 Pozzuoli, Italy; 6 Eye Clinic, Multidisciplinary Department of Medical, Surgical and Dental Sciences, University of Campania “Luigi Vanvitelli”, 81031 Naples, Italy

**Keywords:** *ABCA4*, Stargardt disease, structural variants, CNV

## Abstract

*ABCA4* is the most frequently mutated gene leading to inherited retinal disease (IRD) with over 2200 pathogenic variants reported to date. Of these, ~1% are copy number variants (CNVs) involving the deletion or duplication of genomic regions, typically >50 nucleotides in length. An in-depth assessment of the current literature based on the public database LOVD, regarding the presence of known CNVs and structural variants in *ABCA4*, and additional sequencing analysis of *ABCA4* using single-molecule Molecular Inversion Probes (smMIPs) for 148 probands highlighted recurrent and novel CNVs associated with *ABCA4*-associated retinopathies. An analysis of the coverage depth in the sequencing data led to the identification of eleven deletions (six novel and five recurrent), three duplications (one novel and two recurrent) and one complex CNV. Of particular interest was the identification of a complex defect, i.e., a 15.3 kb duplicated segment encompassing exon 31 through intron 41 that was inserted at the junction of a downstream 2.7 kb deletion encompassing intron 44 through intron 47. In addition, we identified a 7.0 kb tandem duplication of intron 1 in three cases. The identification of CNVs in *ABCA4* can provide patients and their families with a genetic diagnosis whilst expanding our understanding of the complexity of diseases caused by *ABCA4* variants.

## 1. Introduction

*ABCA4* is the most frequently mutated gene leading to inherited retinal disease (IRD) with over 2200 pathogenic variants reported to date [1]. Variation in the *ABCA4* gene is the underlying cause of a spectrum of disorders, which include autosomal recessive Stargardt disease (STGD1), cone-rod dystrophy and late-onset macular dystrophy, collectively known as *ABCA4*-associated retinopathies [2]. The progressive vision loss that affects patients with *ABCA4*-associated retinopathy is caused by dysfunction of the ABCA4 (ATP-binding cassette subfamily A member 4) protein [3]. Alterations of its physiological function within the visual cycle cause an accumulation of toxic retinoid compounds, which leads to the death of photoreceptor and retinal pigment epithelium cells [4,5]. *ABCA4*-associated retinopathy cases are genetically explained by (bi-allelic) single nucleotide variants (SNVs) in the coding regions of the gene or intronic variants affecting splicing (~10%) [6]. Structural variants (SVs) are reported in a small percentage of cases (1%), with the majority being instances of the loss (deletion) or gain (duplication) of genomic material, collectively known as copy number variants (CNVs), in which the number of copies of the corresponding genomic sequence can vary [7,8,9,10,11,12]. Other types of SVs include inversions, insertions and translocations. Deletions and duplications can encompass DNA segments typically ranging from more than 50 nucleotides (nt) up to several megabases (Mb) in size [13,14,15]. CNVs can have no phenotypic effect, existing as an inter-individual genetic variation, or may result in a range of functional implications in human disease, for example, by inducing the gain or loss of function or disrupting regulatory sequences [16,17]. CNVs can prove difficult to detect and characterize using the most common diagnostic genetic tools, such as whole exome sequencing (WES) or gene panels, as the breakpoints of the alterations are frequently located in non-coding regions of the genome [18], although integration with a multiplex ligation-dependent probe analysis can help address this drawback [11,19,20,21]. Nevertheless, several recurrent pathogenic CNVs have been described in *ABCA4*, such as a partial deletion of exon 6 in 12 Spanish affected individuals [12,22,23,24] and a frequent exon 20 to exon 22 deletion, which has been reported in over 26 probands [8,10,21,25,26]. Additionally, the implementation of other sequencing approaches, such as whole genome sequencing (WGS) or whole *ABCA4*-targeted sequencing, permits for the more precise identification of the putative breakpoints of CNVs, ultimately facilitating their validation [12,22,27,28].

In this study, we present an overview of CNVs reported in *ABCA4* to date and report the identification of 7 novel and 8 recurrent CNVs in a total of 21 alleles in previously unsolved *ABCA4*-associated retinopathy cases, which were analyzed by sequencing the entire *ABCA4* gene locus using single-molecule Molecular Inversion Probes (smMIPs).

## 2. Results

### 2.1. Characterization of CNVs in ABCA4-Associated Retinopathies

**Figure 1:**
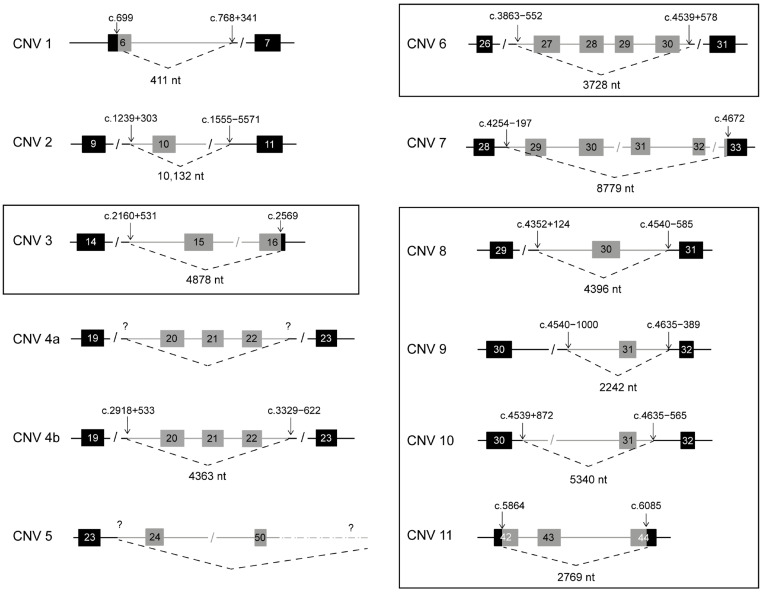
Deletions identified in probands sequenced by smMIPs. In each schematic, intronic regions are represented as horizontal lines and exons are as black boxes. In light grey are represented deleted regions, including partial exon deletions. Black box frames highlight novel deletions. Question marks indicate uncharacterized breakpoints. CNV 1: c.699_768+341del, CNV 2: c.1239+303_1555-5571del, CNV 3: c.2160+531_2569del, CNV 4a: c.(2918+757_2918+981)_(3329-420_3329-644)del, CNV 4b: c.2918+533_3329-622del, CNV 5: g.(?_94458389)_(94505684_94506764)del, CNV 6: c.3863-553_4539+578, CNV 7: c.4254-197_4672delinsGCTTTTT, CNV 8: c.4352+123_4540-585del, CNV 9: c.4540-1000_4635-389delinsTGCCCG, CNV 10: c.4539+872_4635-565delins28 and CNV 11: c.5864_6085del.

**Figure 2:**
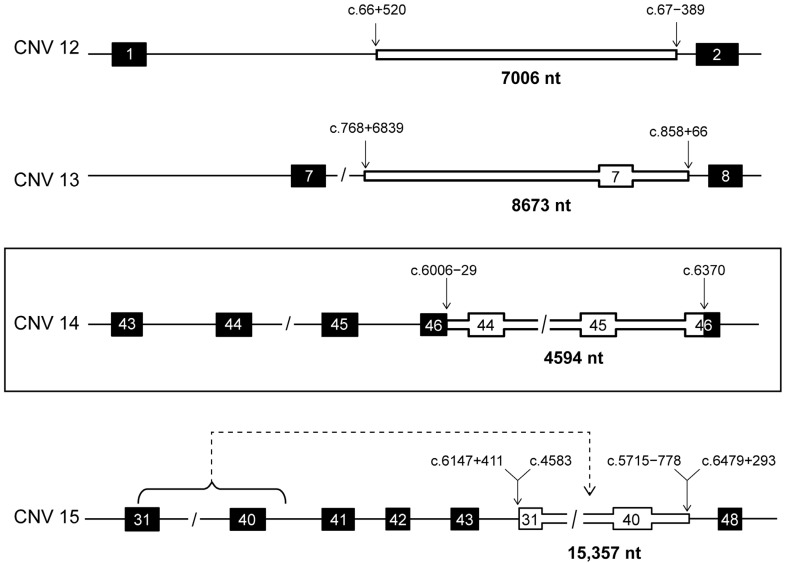
Duplications and complex rearrangement identified in probands sequenced by smMIPs. In each schematic, intronic regions are represented as horizontal lines and exons are as black boxes. Duplicated regions are depicted as bold, white-filled sections. Black box frames highlight the novel duplication. The stapled line arrow in CNV 15 highlights the downstream insertion of the duplicated region. CNV 12: c.66+520_67-389dup, CNV 13: c.768+6839_858+66dup, CNV 14: c.6006-29_6370dup and CNV 15: c.6147+411_c.6479+293delins4583_5715-778.

An analysis of the smMIP sequencing data was performed for 147 *ABCA4*-associated retinopathy probands. Of these, 15 probands were suspected to carry a CNV as a second allele, with at least one other variant in *ABCA4* identified using a previous screening method (Table S1). A coverage depth analysis led to the detection of 15 different CNVs in a total of 20 alleles, including 6 novel deletions and 1 novel duplication (Figure 1 and Figure 2 and Table 1). Sanger sequencing facilitated the identification of the exact CNV junction(s) in 17 probands (Figures S1–S14). A total of 19 cases were considered solved by the identification of a CNV as the second allele.

### 2.2. Deletions

In 13 sequenced probands, 12 different exon-spanning deletions were identified, the majority of which were previously reported in the literature. The most frequent deletion in the cohort (n = 2) was a deletion of intron 19 to intron 22 (CNV 4 in Figure 1), which was detected by a coverage depth analysis and confirmed by a visualization of the BAM files in Integrative Genomics Viewer (IGV) version 2.4. The position of the predicted breakpoints could be confirmed in only one proband (Figure S4), resulting in the identification of a deletion of 4363 nt (c.2918+533_3329-622del) with the breakpoints differing slightly from two previous reports [10,22]. In the remaining case, the Sanger sequencing of the junction PCR products failed. Similarly, the exact junctions of CNV 5, a deletion spanning exon 24 to the 3′ UTR of *ABCA4*, could not be characterized as the position of the downstream breakpoint falls outside of the region targeted by smMIPs.

A short partial deletion of exon 6, c.699_768+341del (CNV 1 in Figure 1), was identified in two probands, corresponding to the second most frequent deletion in *ABCA4* (12 cases reported in LOVD, Table S2), which is particularly frequent in patients of Spanish origin [12,22,23,24]. Deletion c.1239+303_1555-5571del (CNV 2 in Figure 1) encompasses 10,132 nt from intron 9 to intron 10, and has marginally different breakpoints compared to a previously reported case (c.1239+291_1555-5574del) [10], but was not considered a novel deletion. The last known deletion detected was CNV 7 (c.4254-197_4672delinsGCTTTTT), which spans a region from intron 29 to the first 5 nt of exon 33 with a 7 nt insertion at the CNV junction, corresponding to a previously reported CNV [12].

Six novel deletions were detected and validated in separate probands. CNV 3, c.2160+531_2569del, spans 4878 nt from intron 15 to the last 18 nt of exon 16. Similarly, CNV 11 (c.5864_6085del) exhibited both proximal and distal breakpoints within the exonic regions, specifically in exon 42 and exon 44, respectively. The four remaining novel deletions (CNVs 6, 8, 9 and 10; Figure 1) all exhibit one of the two breakpoints in intron 30. In detail, CNV 6 is a deletion of 3728 nt from intron 26 to intron 30, while CNV 8 is a deletion of 4396 nt spanning exon 30. Both CNV 9, c.4539+872_4635-565delins28, and CNV 10, c.4540-1000_4635-389delinsTGCCCG, are deletions encompassing exon 31. Nevertheless, their lengths (2242 nt and 5340 nt, respectively) and breakpoints are distinct; hence, each was considered a different defect.

### 2.3. Duplications and Complex Rearrangement

Three distinct duplications were identified in a total of five probands, and one complex rearrangement was revealed in one proband (Figure 2). An intronic duplication encompassing 7006 nt of intron 1 (c.66+520_67-389dup) (CNV 12) was identified in three cases: two unrelated probands submitted from Italy and one proband recruited from Slovenia. CNV 13 is an 8673 nt duplication encompassing exon 7, c.768+6839_858+66dup, which had been previously reported [22], while CNV 14 is a novel defect consisting of a 4594 nt duplication from intron 44 to exon 46 (c.6006-29_6370dup) (Figure 2). Finally, a complex rearrangement (CNV 15) was identified in one proband. The coverage depth analysis highlighted the presence of a duplication of the region from intron 31 to intron 41 and a downstream deletion from intron 43 to 47. A junction PCR analysis was performed in a semi-blind manner, i.e., by a multiplex PCR with primers binding the regions around the expected breakpoints of both the deletion and duplication. This enabled the detection of a 15,357 nt insertion spanning from the middle of exon 31 to intron 44 (c.4583_5715-778) that was inserted at the junction of a deletion encompassing intron 44 through intron 47 (2725 nt) (Figure 2 and Figure S14). This result suggests that the duplication and the downstream deletion described did not occur as separate events but as one unique rearrangement, consisting of the insertion of the duplicated region in the position of the deleted downstream region; hence, the defect can be defined as a deletion-insertion (delins) (c.6147+411_c.6479+293delins4583_ 5715-778).

### 2.4. Microhomology and Repetitive Elements

Where possible, an analysis of the CNV breakpoint junction sequences, together with the proximal and distal regions around the breakpoints, was performed to identify the regions of microhomology, potential repetitive elements and DNA motifs underlying the formation of the CNVs (Table S3 and Figure S15). The proximal and distal sequences of CNV 4b and CNV 6 showed the highest homology due to the presence of *Alu* repeats at the breakpoints. The repetitive elements in CNV 4b are consistent with previous reports (AluSx at the proximal and AluY at the distal breakpoint), while in CNV 6 the proximal region carries AluSc8 and the distal region AluSz6. In light of these results, these deletions are likely to be *Alu/Alu*-mediated rearrangements. In the majority of other cases, short microhomologies were observed at the junctions, ranging from 1 to 7 nt, with the exception of CNV 7, where no microhomology was present. On the other hand, in CNV 7 as well as in CNV 9 and CNV 10, insertions of short random sequences (6–28 nt) were present at the breakpoint junction; these could be information scars that derived from the action of repair mechanisms, such as non-homologous end joining or microhomology-mediated end joining. Repetitive elements were identified spanning the breakpoints of six CNVs, including the aforementioned CNV 7. In the large intron 1 duplication, both the proximal and distal regions correspond to DNA transposons (MER104 and MER5A1, respectively). The remaining repetitive elements detected were one (SINE) and two long interspersed nuclear elements (LINEs). The most frequent DNA motif detected was the Oligo(G)n tracts (22 potential motifs), potentially leading to a tetraplex structure formation, spanning a CNV breakpoint in three instances. Other non-B DNA motifs identified were short tandem repeats (n = 3) leading to single-strand loops or hairpins, inverted repeats (n = 2) leading to cruciform DNA conformation, one mirror repeat leading to a triple-helical DNA structure and one Z-DNA motif. Of these, only the mirror repeat was directly overlapping the CNV breakpoint (CNV 15) (Table S3).

### 2.5. Literature-Reported CNVs in ABCA4

**Figure 3:**
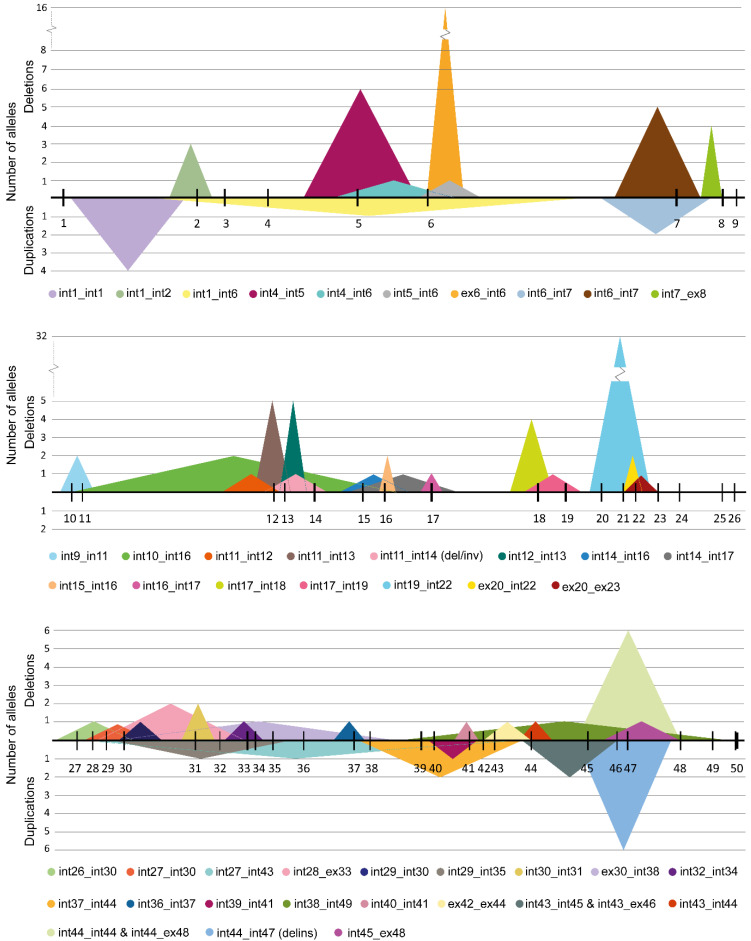
The landscape of the reported structural variants in *ABCA4.* All the CNVs submitted to LOVD and identified in this study are depicted as triangles encompassing the affected region of *ABCA4*. The *ABCA4* gene sequence has been divided into three sections (top: exon 1 to exon 9, middle: exon 10 to exon 26 and bottom: exon 27 to exon 50), and the numbered vertical lines represent the exons. For each section, the peaks above and below represent the deletions and duplications, respectively. The height of the peaks corresponds to the number of alleles reported in LOVD. Below each section, the color legends for all the CNVs show the details of the intronic or exonic region of the breakpoints. Complex defects, such as deletion-inversions and deletion-insertions, are annotated as del/inv and delins, respectively, in the legend. Defects with one or both breakpoints in the intergenic regions are not included.

Overall, with the novel CNVs identified in this study, there are 70 different SVs reported to affect *ABCA4*, including two whole-gene deletions and seven deletions where only one breakpoint falls within the gene (Table S2). The majority are deletions, with 55 variants for a total of 126 cases, while duplications accounted for 9 different defects (13 reports) and other complex variants for 4 rearrangements, including deletion-inversions, delins and duplication-inversions (9 reports). Deletions are distributed over most of the gene, while the majority of duplications (n = 6/9) are located in the second half of the gene, between intron 27 and intron 49 (Figure 3). Interestingly, no CNV affecting exons 24 to 26 has been yet reported. In ~40% of cases (n = 26/70), the exact breakpoints were not reported, as the sequencing techniques used did not permit their precise identification. In this study, CNVs with defined breakpoints were counted as separate events from uncharacterized defects. Nevertheless, the possibility that a portion of these CNVs share the same breakpoints as other reports cannot be dismissed and it is thus likely that the number of unique events is being overestimated in this study.

## 3. Discussion

*ABCA4* whole-gene smMIP-based sequencing was used to analyze 147 *ABCA4*-associated retinopathy probands in search of causative CNVs. While the occurrence of CNVs in *ABCA4* is relatively low (~1% of all variants) compared to the overall incidence in IRD cohorts (~7–9%) [18,28,29], the number of reported CNVs in the gene continues to steadily increase. In 2020, the number of known SVs was 46 in a total of 92 alleles [6]. The results of this study, both the experimental ones and from the LOVD search, uncovered 60 CNVs in a total of 131 alleles. While a direct comparison with previous studies is not possible due to the different inclusion criteria (Cremers et al., 2020 [6], CNV > 20 nt; this study, CNV > 50 nt), these numbers clearly show that the search and analysis of potential CNVs in *ABCA4* remains essential to genetically solve many probands. Despite the majority of CNVs being unique events, there are regions in *ABCA4* that appear to be more prone to their formation, leading to recurrent events. Additionally, the absence of a precise breakpoint analysis in a large portion of reports constitutes a challenge in the estimation of the effective number of unique and recurrent CNVs. The most widely used diagnostic sequencing techniques, such as WES or gene panel (exonic) sequencing, highlight the presence of deleted or duplicated exons but are not effective in the detection of the precise location of the defects. On the contrary, the high whole-gene coverage obtained with the smMIPs in this study permitted the efficient identification of eight recurrent and seven novel CNVs and, in the majority of the cases, facilitated the characterization and validation of the exact breakpoints.

Eight of the eleven detected deletions are out of frame and predicted to be deleterious (Table 1), although the precise effect of the variants at the RNA and protein level has not been functionally analyzed. CNVs 2 and 11 both maintain the reading frame; nevertheless, they are both likely to act as severe alleles as they disrupt the structure and function of the protein. In particular, the deletion of exons 10 and 11 (CNV 2) affects the region encoding part of the extracellular domain 1 of ABCA4 and contains several glycosylation positions involved in the recognition of the transported substrate [30,31]. Previous studies have suggested that the deletion of these in-frame exons is likely to result in severe or deleterious alleles [32]. CNV 11 is also an in-frame deletion, but both breakpoints fall in the exonic regions such that a new “fusion exon” composed of the start of exon 42 and the end of exon 44 would be created, likely affecting the folding and protein function. Additionally, these exons translate to the nuclear binding domain 2 of ABCA4, which is involved in the ATP-hydrolysis activity of the protein [30,31]. Finally, the effect of CNV 5 is unclear, as the downstream breakpoint, outside of *ABCA4*, is unknown. Nevertheless, in the majority of cases, a segregation analysis is needed to confirm that the novel findings are not on the same allele as previously reported pathogenic variants. Of the three duplications, only CNV 14 disrupts the open reading frame; the exon 7 duplication (CNV 13) is in-frame while CNV 12 is the only completely non-coding defect detected, encompassing almost the entire intron 1 of *ABCA4*. The effect of the CNV 12 duplication remains unclear, although it could be altering RNA splicing or, as it has been previously suggested, disrupting transcription factor binding sites present within the region [10]. While outside the scope of this study, further investigation of the functional effect of this duplication, that spans almost the entire intron 1, is needed to understand its impact on *ABCA4* RNA. The deletion of exon 20 to 22 is by far the most frequent CNV in *ABCA4* (29 reports, Table S2 and Figure 3). This was also reflected in the present study, where two additional probands carrying the deletion were identified, although only in one case the exact CNV junction could be determined. Interestingly, the breakpoints of the novel report and those of the previous reports (two cases with defined breakpoints) [10,22] are all slightly different; nevertheless, in all the cases, the proximal and distal breakpoint regions fall on the same *Alu* repeat elements (AluSx and AluY, respectively), with over 80% of the sequence identity between the consensus sequence of the two elements. The high sequence similarity of the repetitive elements could explain the difficulties in pinpointing the exact breakpoints of the deletion encountered in this study. The presence of the *Alu* repeats at the breakpoints of both characterized deletions suggests these CNVs are generated by *Alu/Alu*-mediated rearrangement mechanisms, implicating replication-based mechanisms, such as fork stalling and template switching (FoSTes) and microhomology-mediated break-induced replication (MMBIR) [33,34], although the involvement of meiotic rearrangement mechanisms, such as non-allelic homologous recombination (NAHR), cannot be completely excluded [35,36]. *Alu/Alu*-mediated rearrangement mechanisms could also explain the non-recurrent deletion of exon 27 to exon 30 (CNV 6), where the repeat elements AluSc8 and AluSz6 were identified at the proximal and distal breakpoints, respectively. Interestingly, the final CNV with repetitive elements on both breakpoints was the intron 1 duplication (CNV 12), displaying the MER104 consensus sequence at the proximal and MER5A1 at the distal breakpoint, both of which are DNA transposons. While these elements do not show any sequence identity, their presence could indicate genome instability within the region.

The complex CNV 15 was previously reported in five affected individuals of a large consanguineous Tunisian family [37]. In this study, the CNV was identified in a proband of north African descent, suggesting that this variant could be a founder mutation and more frequent than currently reported in the literature. The variant, which was initially thought to involve two concurrent but separate events, i.e., a duplication of exon 31 through intron 40 and a deletion of intron 45 through 47, was shown to be a complex rearrangement consisting of the insertion of the duplicated region at the junction of the downstream deleted region of *ABCA4*. Even though the detection and characterization of this complex defect was possible in this study, smMIP locus sequencing remains an imperfect method for the identification of more complex rearrangements, as well as non-CNV SVs, such as inversions and translocations. The majority of reported *ABCA4* SVs are intragenic (micro-)CNVs, spanning regions between 400 and 27,000 nt, with the breakpoints contained in the gene sequence itself. Nevertheless, seven CNVs where one or both breakpoints are located outside of the gene, including two reports of larger chromosomal deletions spanning over the whole *ABCA4* locus, are known in the literature. While these appear to be rare events, it is possible that they remain undetected more frequently, because targeted sequencing methods, such as the one utilized in this study, would fail to highlight them as easily as intragenic defects. The detection of SVs such as inversions, insertions and translocations requires the implementation of alternative sequencing approaches covering the complete genome. “Classical” short-read WGS can be a good alternative, as it reports information for the whole genome. On the other hand, this technique, as with all short-read technologies, carries considerable limitations in the resolution of highly repetitive sequences, which are frequently involved in the formation of SVs [38,39]. In recent years, several novel strategies have been developed based on amplification-independent techniques able to effectively screen repetitive regions of the genome. Some notable examples are single-molecule real-time (SMRT) sequencing by Pacific Biosciences (PacBio) [40,41,42] and nanopore sequencing by Oxford Nanopore Technologies (ONT) [43,44], based on long-read sequencing, as well as optical genome mapping, which utilizes restriction enzyme patterns to identify large SVs [45,46]. The implementation of these novel approaches might lead to the identification of novel (likely rare) SVs, which have, to date, been missed in *ABCA4*-associated retinopathy cases.

In conclusion, with this study, we identified and characterized seven novel rearrangements and provide an updated overview of the known CNVs detected in *ABCA4*, highlighting the relevance of CNV screening to reach a genetic diagnosis in unsolved cases and how smMIP sequencing is a good approach for the precise identification of (micro-)CNVs affecting the *ABCA4* locus. Overall, this study broadens the understanding of the role of SVs, and especially CNVs, in *ABCA4*.

## 4. Materials and Methods

### 4.1. Sample Origin and Sequencing

A total of 147 patients underwent sequencing of the complete *ABCA4* gene locus using a novel smMIP platform. A total of 2973 smMIPs were designed by Molecular Loop Biosciences, Inc. (Woburn, MA, USA) incorporating the entire *ABCA4* gene (NM_000350.3; encompassing GRCh37/hg19 chr1:94458507-94588844). Each smMIP targets a 225 nucleotide (nt) capture region using a 5′ extension and a 3′ ligation probe arm, flanked by dual custom index adapter sequences, two index primer sequences of 10 nt in length and dual 5 nt randomers. The design enabled the distribution of smMIPs across *ABCA4* where each nucleotide position in the gene was covered, on average, by five probes (Figure S16). DNA libraries were prepared as previously described [47] and library pools were sequenced by paired-end sequencing on a NovaSeq 6000 (Illumina, San Diego, CA, USA) platform using SP reagent kits v1.5 (300 cycles) with custom sequencing primers. The majority of the included probands (103/147) had been previously sequenced using an alternative smMIP gene panel but remained genetically unsolved with only one pathogenic *ABCA4* allele identified. More precisely, 60 probands were sequenced using a macular degeneration (MD) smMIP platform [47] and 43 with a retinitis pigmentosa (RP) and Leber congenital amaurosis (LCA) smMIP panel [48]. The remaining forty-four samples had been previously sequenced, in a diagnostic setting, by alternative methods, i.e., clinical WGS, next-generation sequencing target panel, Sanger sequencing or dHPLC, which had identified one or more causal *ABCA4* variants. These cases, which included 13 samples carrying suspected CNVs, were selected for whole *ABCA4* smMIP sequencing, because the identified variants were not sufficient to consider them genetically solved. All the probands had undergone clinical assessment in the respective center of origin and were included in this study due to the presence of one pathogenic variant in *ABCA4* or due to the presence of *ABCA4*-associated retinopathy in their differential diagnosis. All the samples were collected according to the tenets of the Declaration of Helsinki and written informed consent was obtained for all the probands participating in the study. The Medisch Ethische Toetsings Commissie Erasmus MC gave approval to conduct these studies (MEC-2010-359). The DNA was diluted to 15–25 nanograms per microliter (ng/μL) and the DNA integrity was assessed by agarose gel electrophoresis.

### 4.2. CNV Analysis

The detection of CNVs was performed using an Excel (Office version 365) script developed in-house based on the smMIPs read depth, as previously described [12]. Briefly, the read number of all the smMIPs was normalized in each sample to the total number of reads in the proband, followed by a second step in which the coverage of each probe in a specific sample was compared to the average coverage across all the probands in the sequencing run, obtaining a final normalized value between 0 and 2. The CNV detection was facilitated by the conditional formatting of the output to highlight the following ranges: <0.3, homozygous deletion; between 0.3 and 0.7, heterozygous deletion; between 1.2 and 1.7, gain of one copy; and >1.7, gain of two or more copies. Values between 0.8 and 1.2 were considered indicative of the absence of CNVs. An average smMIP coverage lower than 25x was considered too low to confidently call CNVs; therefore, samples with an average read coverage below this threshold were excluded from the analysis. In parallel to the CNV script analysis described above, all the samples were screened for deletions, duplications and insertions of 50–100 nt in length using an in-house annotation pipeline [49]. In line with the current consensus, variants less than 50 nt in length were not considered as CNVs but as small insertions or deletions (indels) [50,51].

### 4.3. CNV Validation and Breakpoint Analysis

The presence of all the CNVs identified through the coverage script screening were visualized in the proband binary alignment map (BAM) file to verify their presence. Subsequently, the region containing the expected CNV was amplified by polymerase chain reaction (PCR) using AmpliTaq Gold DNA polymerase (Thermofisher Scientific, Waltham, MA, USA) with an input of 30 ng of genomic DNA. The following protocol was used: 94 °C for 5 min, 35 cycles of 94 °C for 30 s, 58 °C for 30 s, 72 °C for 3 min and a final elongation of 72 °C for 5 min. The elongation times were adjusted according to the calculation of 1 min per kb, when necessary. After confirmation of amplification by gel electrophoresis, the PCR product was purified from an agarose gel slice (NucleoSpin PCR clean-up kit, Macherey-Nagel, Düren, Germany) or by ExoSAP (ExoI and FastAP from Thermo Fisher Scientific, Waltham, MA, USA) and underwent Sanger sequencing to identify the exact junction of the CNV. The primers used for the junction PCR in each sample are reported in Table S4.

The microhomology at the breakpoints of each CNV was assessed using the multiple alignment tool ClustalW (https://www.genome.jp/tools-bin/clustalw, accessed on 27 March 2024) [52] on a region of 150 nt flanking the 5′ and 3′ breakpoints of the deletion or duplication (Table S3 and Figure S15). The latter were further analyzed for the presence of (a) repetitive elements using Repeat Masker (https://www.repeatmasker.org/, accessed on 27 March 2024) [53] and Censor (https://www.girinst.org/censor/, accessed on 27 March 2024) [54]; (b) non-B motifs, such as direct, inverted, mirror and GT repeats, using nBMST (https://nonb-abcc.ncifcrf.gov/apps/nBMST/default/, accessed on 27 March 2024) [55]; and (c) quadruplex-forming G-rich sequences using QGRS Mapper (https://bioinformatics.ramapo.edu/QGRS/analyze.php, accessed on 27 March 2024) [56] (Table S3 and Figure S15). In the case that both the proximal (5′) and distal (3′) breakpoints presented repetitive elements, the sequence identity between their consensus sequences, obtained from Dfam (https://www.dfam.org/home, accessed on 27 March 2024) [57], was assessed using BLAST2 (https://blast.ncbi.nlm.nih.gov/Blast/, accessed on 27 March 2024) [58].

### 4.4. CNV Literature Selection

A comprehensive overview of the CNVs in *ABCA4*, reported to date, was compiled from the available literature by searching the public database LOVD (http://www.lovd.nl/ABCA4; accessed on 28 September 2023). The following terms were queried in the database to highlight the structural variants: contains “dup”, contains “del”, contains “inv”, contains “ins”. This list was curated to remove duplicated reports and exclude short variations under 50 nt, e.g., the frequent 23 nt deletion c.4254-37_4254-15del. Where required, genomic and cDNA notations were corrected to adhere with ACMG guidelines to obtain a final comprehensive list of uniquely reported CNVs in *ABCA4*.

## Figures and Tables

**Table 1:** Details of identified copy number variants (CNVs) after smMIP sequencing. Novel CNVs are reported in bold text. Del: deletion, dup: duplication, IN: in frame, ins: insertion, n/a: not applicable, OUT: out of frame, and #: number of.

CNV	Type	Region	IN/OUT	# Alleles	cDNA Variant	Protein Variant	Genomic Variant (hg19)	Size (nt)
1	del	ex6_int6	OUT	2	c.699_768+341del	p.(?)	g.94564009_94564419del	411
2	del	int9_int11	IN	1	c.1239+303_1555-5571del	p.(Ala414_Glu518del)	g.94534444_94544575del	10,132
**3**	del	**int14_ex16**	**OUT**	**1**	**c.2160+531_2569del**	p.(?)	**g.94520685_94525562del**	**4878**
4a	del	int19_int22	OUT	1	c.(2918+757_2918+981)_	p.(Ser974Glnfs*64)	g.(94507378_94507602)_	n/a
					(3329-420_3329-644)del		(94511494_94511718)del	
4b	del	int19_int22	OUT	1	c.2918+532_3329-622del	p.(Ser974Glnfs*64)	g.94507580_94511943del	4363
5	del	int23_ex50	n/a	1	n/a	p.(?)	g.(?_94458389)_(94505684_94506764)del	n/a
**6**	del	**int26_int30**	**OUT**	**1**	**c.3863-553_4539+578del**	p.(Gly1288Glufs*41)	**g.94494423_94498152del**	**3728**
7	del	int28_ex33	OUT	1	c.4254-197_4672delinsGCTTTTT	p.(?)	g.94487503_94496279delinsAAAAAGC	8779
**8**	del	**int29_int30**	**OUT**	**1**	**c.4352+123_4540-585del**	p.(Tyr1453Hisfs*11)	**g.94491189_94495861del**	**4396**
**9**	del	**int30_int31**	**OUT**	**1**	**c.4540-1000_4635-389delinsTGCCCG**	p.(Arg1514Leufs*9)	**g.94489363_94491604delisCGGGCA**	**4591**
**10**	del	**int30_int31**	**OUT**	**1**	**c.4539+872_4635-565delins28**	p.(Arg1514Leufs*9)	**g.94489539_94494129delins28**	**2242**
**11**	del	**ex42_ex44**	**OUT**	**1**	**c.5864_6085del**	**p.(?)**	**g.94471059_94473825del**	**2469**
12	dup	int1_int1	IN	3	c.66+520_67-389dup	p.(?)	g.94579011_94586016dup	7006
13	dup	int6_int7	IN	1	c.768+6839_858+66dup	p.(Leu257_Glu286dup)	g.94548842_94557511dup	8670
**14**	dup	**int43_ex46**	**OUT**	**1**	**c.6006-29_6370dup**	**p.(?)**	**g.94466574_94471167dup**	**4594**
15	ins	ex31_int40/	OUT	2	c.6147+411_6479+293delins4583_5715-778	p.(?)	g.94466098_94470586delins	15,357
		int44_int47					94475205_94490561	

## Data Availability

The original contributions presented in the study are included in the article/Supplementary Materials, further inquiries can be directed to the corresponding author.

## Supplementary Materials

The supporting information can be downloaded at: https://www.mdpi.com/article/10.3390/ijms25115940/s1.
