# Supplementary material for: Novel and Recurrent Copy Number Variants in ABCA4-Associated Retinopathy
Source: Int J Mol Sci. 2024 May 29;25(11):5940. doi: 10.3390/ijms25115940 (PMC11173210; doi:10.3390/ijms25115940)
Supplement: Supplementary file 1 [file ijms-25-05940-s001.zip › Figures.pdf]

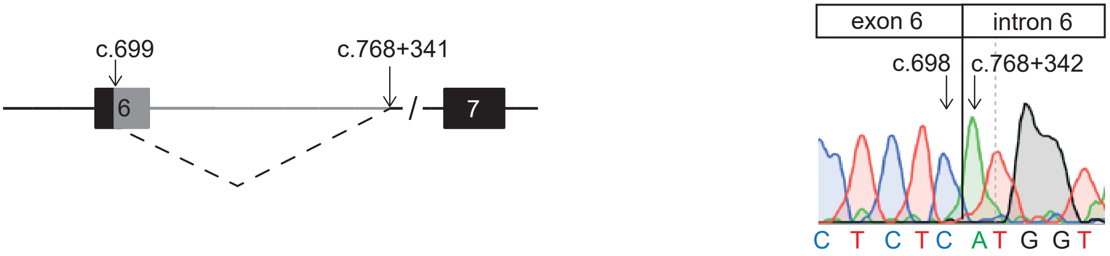

Figure S1: Sanger sequencing validation of CNV 1 identified in samples 079919 and 079921.

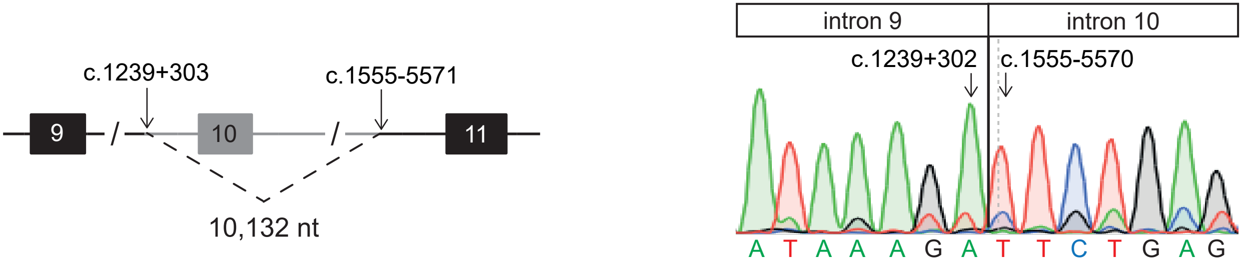

Figure S2 : Sanger sequencing validation of CNV2 identified in sample 079908.

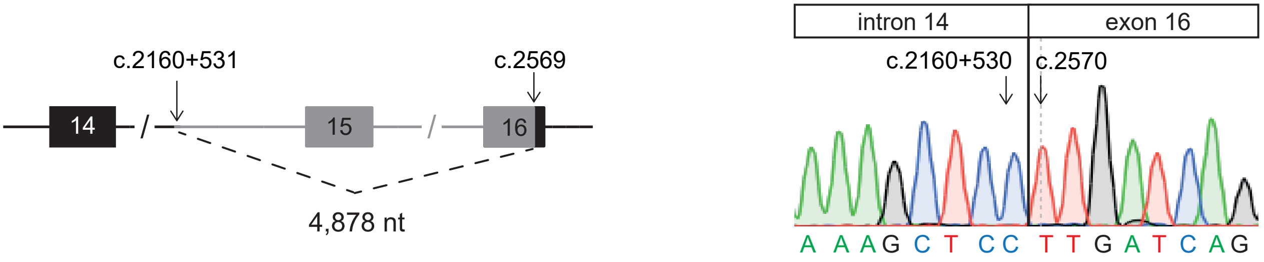

Figure S3: Sanger sequencing validation of CNV3 identified in sample 079929.

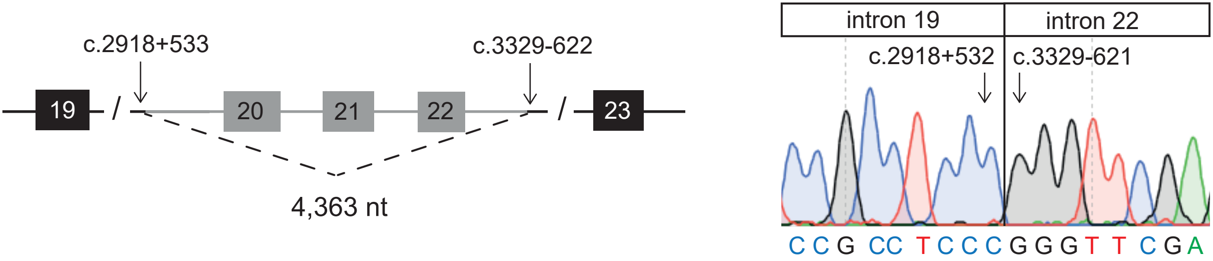

Figure S4 : Sanger sequencing validation of CNV4b identified in sample 079923.

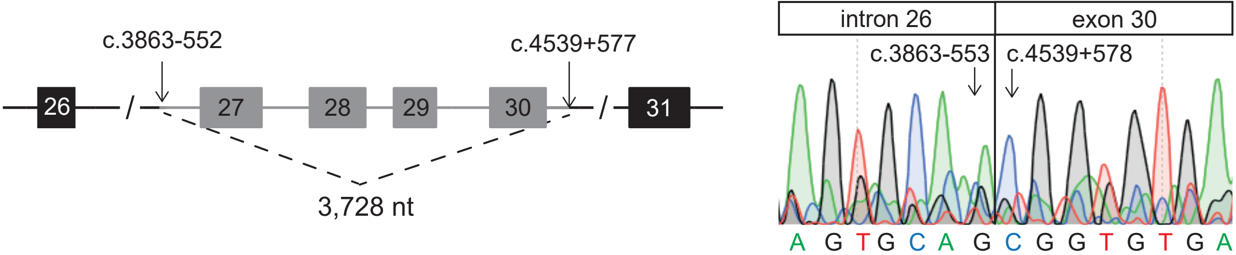

Figure S5: Sanger sequencing validation of CNV6 identified in sample 079932.

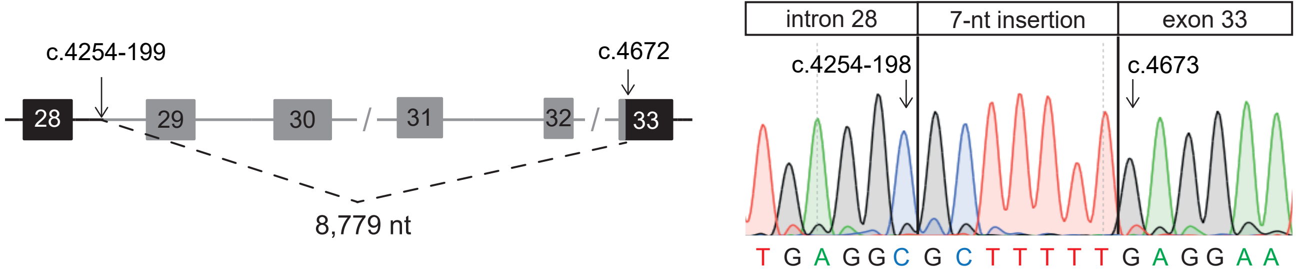

Figure S6: Sanger sequencing validation of CNV7 identified in sample 079924.

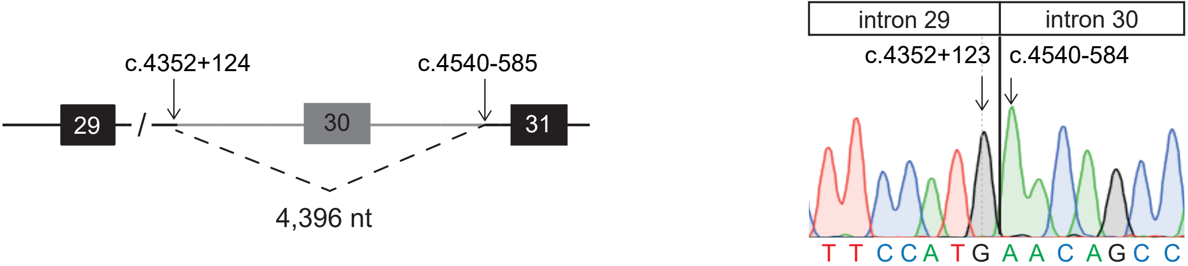

Figure S7: Sanger sequencing validation of CNV8 identified in sample 079927.

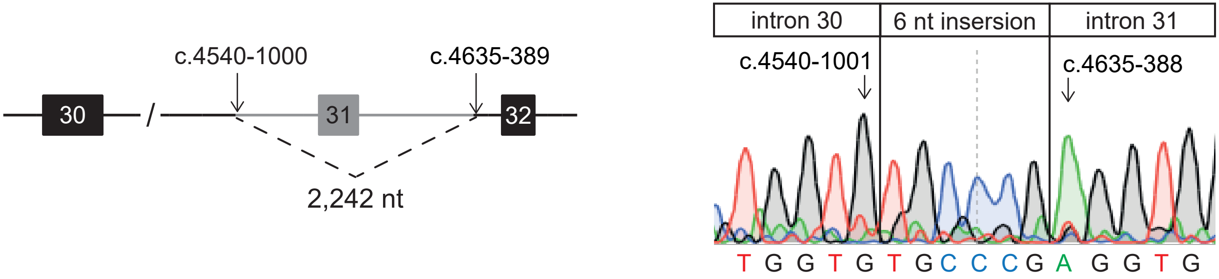

Figure S8: Sanger sequencing validation of CNV9 identified in sample 067513.

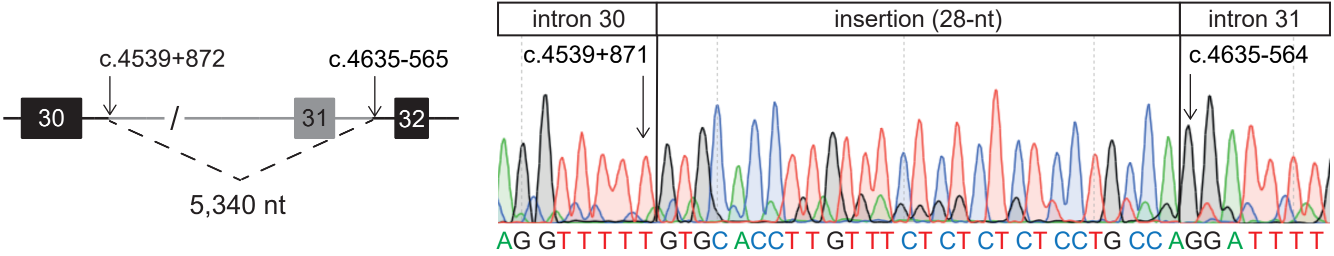

Figure S9: Sanger sequencing validation of CNV10 identified in sample 079925.

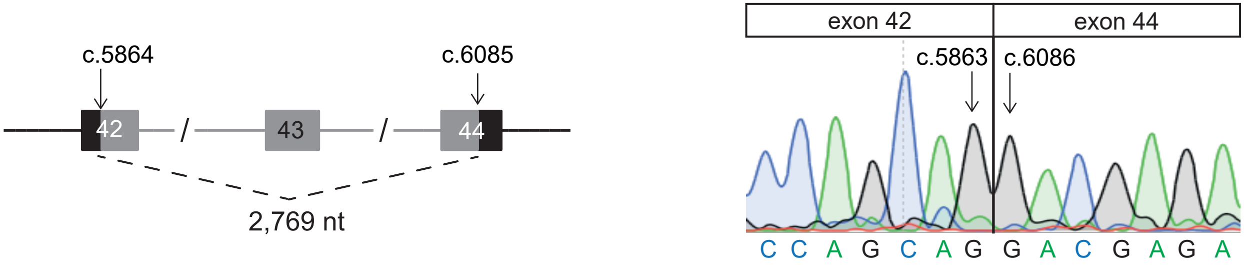

Figure S10: Sanger sequencing validation of CNV11 identified in sample 079922.

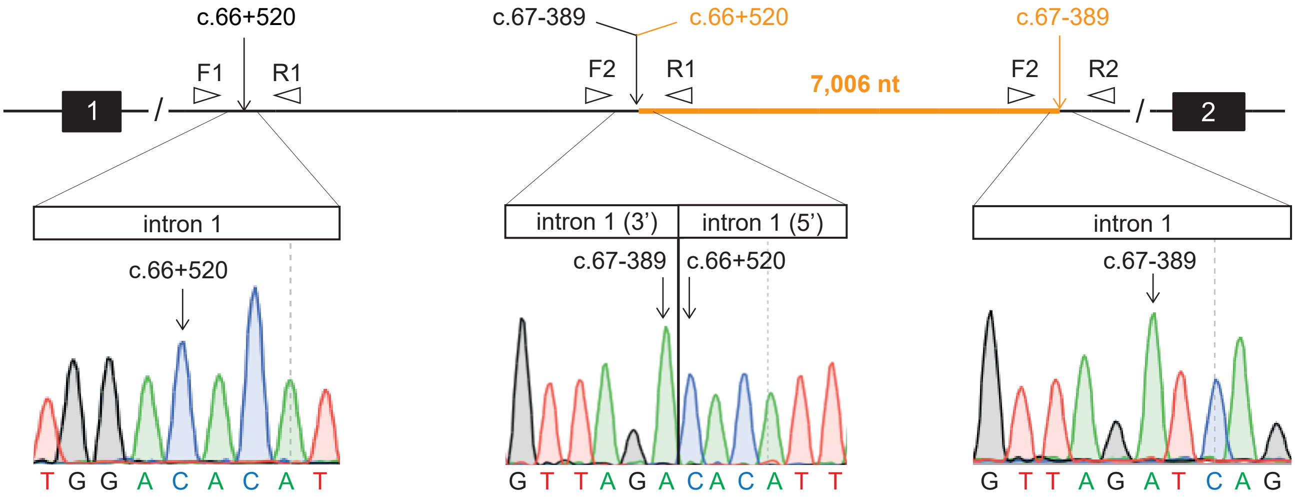

Figure S11: Sanger sequencing validation of CNV12 identified in samples 073768, 075728 and 081420.

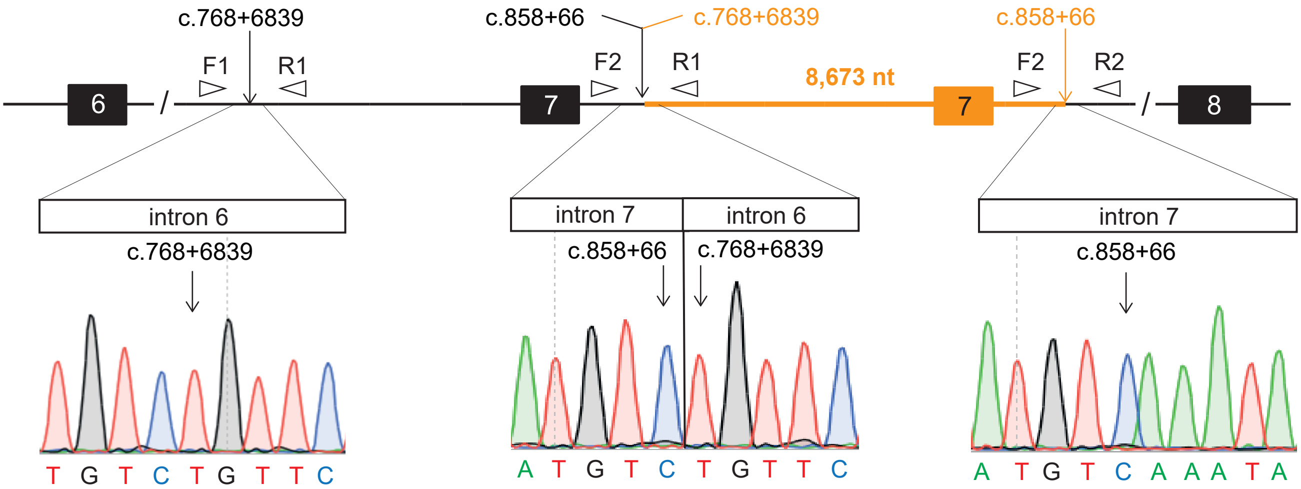

Figure S12: Sanger sequencing validation of CNV13 identified in sample 079926.

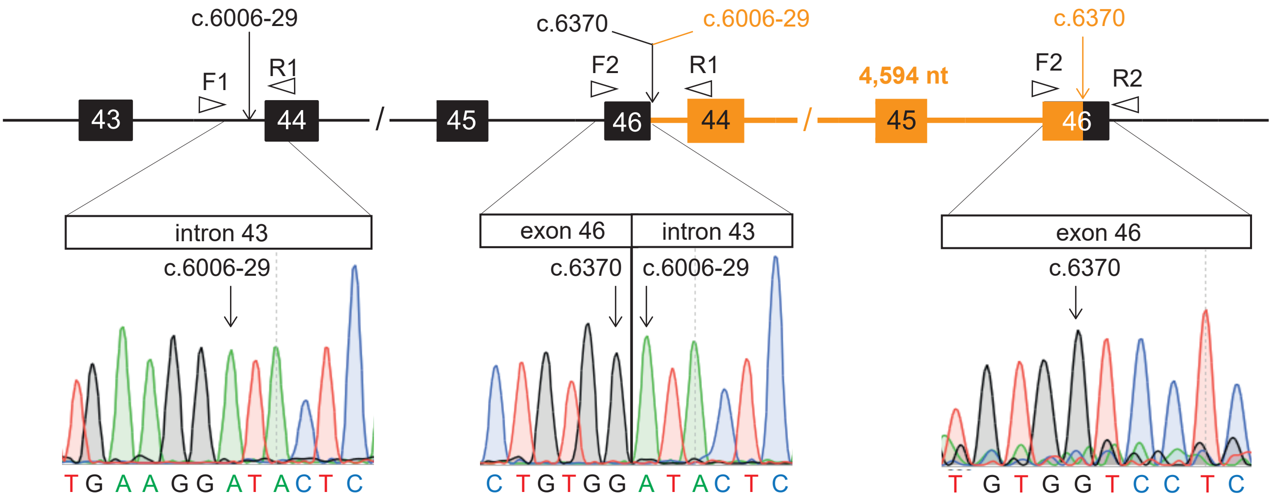

Figure S13: Sanger sequencing validation of CNV14 identified in sample 079920.

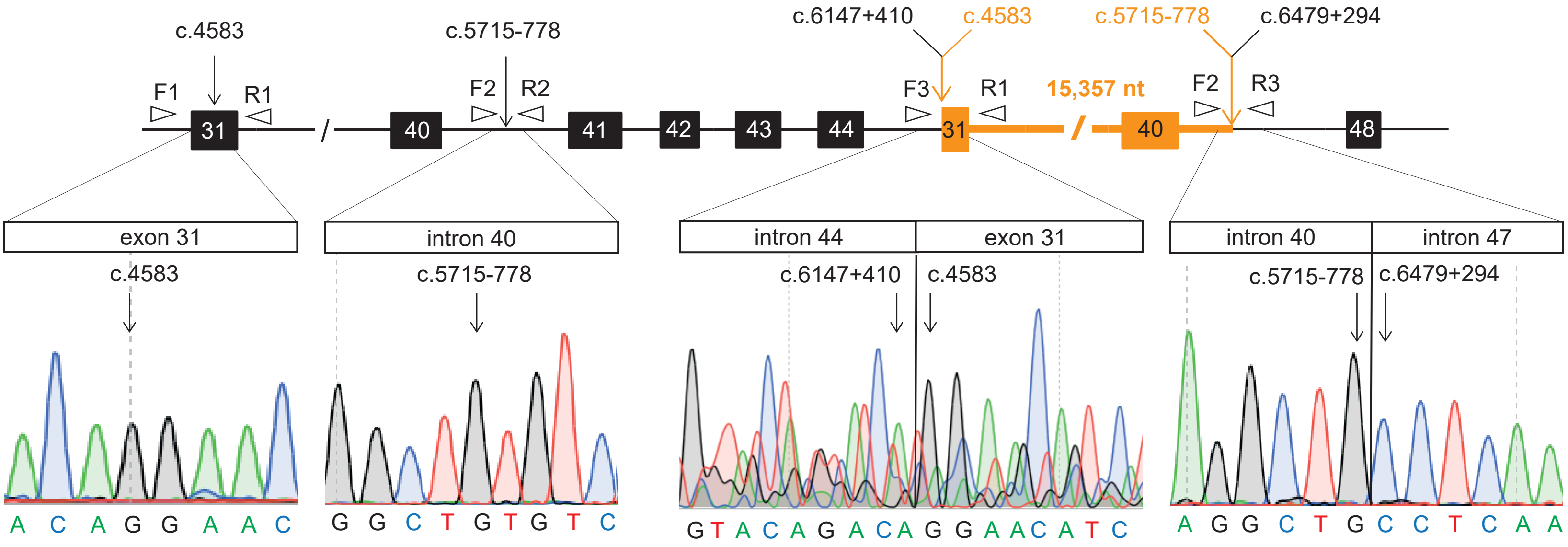

Figure S14: Sanger sequencing validation of CNV15 identified in sample 079930.

CNV 1

c.699_768+341del

proximal	CCTCCTGGAGCGCTTCATCATCTTCAGCCAGAGACGCGGGCAAAGACGG
junction	CCTCCTGGAGCGCTTCATCATCTTCAGCCAGAGACGCGGGCAAAGACGG
distal	TCCTCTAACAGAGTGGTAAAGGAAGGAGGAGGCCTGGTTTGACTCCCTGA

Oligo(G)n tract

Oligo(G)n tract (on complementary strand)

proximal	TGCGCTATGCCCTGTGCTCCCTC**TC**CCAGGGCACCCTACAGTGGATAGAA
junction	TGCGCTATGCCCTGTGCTCCCTC**TC**ATGGTAAGATATTGAACCTTTTCTG
distal	CCTGCTATTTCCTAGCCAGGTGA**TC**ATGGTAAGATATTGAACCTTTTCTG

SINE (MIRb)

proximal	GACACTCTGTATGCCAACGTGGACTTCTTCAAGCTCTTCCGTGTGGTAAG
junction	GTCCCAGTACTCATCTATAAAACAAATATAATACTTTACAGAGTGGTAGG
distal	GTCCCAGTACTCATCTATAAAACAAATATAATACTTTACAGAGTGGTAGG

CNV 2

c.1239+302_1555-5570del

proximal	TCTTTCCCATTGCTTGAATTATATCCCTAATGGAAGGGTTCACAATTCTC
junction	TCTTTCCCATTGCTTGAATTATATCCCTAATGGAAGGGTTCACAATTCTC
distal	AGGGGAGGAGGAAAGCCCTTTCCTTGCTTTATTGTCATTGGCTGTCATAG

Oligo(G)n tract inverted repeat

Oligo(G)n tract

proximal	TGAATCCTGGCTACTCAGATAA**AGA**CAGGGAGGAAGGGAGGAAGGGTATT
junction	TGAATCCTGGCTACTCAGATAA**AGA**TTCTGAGCTCCGGCTATAACATTTG
distal	GGCATTACAATGGTTCTCTTTG**AGA**TTCTGAGCTCCGGCTATAACATTTG

Oligo(G)n tract

proximal	TTCTCCCAGGGGGTCCAAATCTAGCTTTAACGAGGGAGGTTCTGAGAAAA
junction	CCCAGAATCTGCCTCTGAGGCCTTAAGACACTGTGTTTTTATTCAGCAAA
distal	CCCAGAATCTGCCTCTGAGGCCTTAAGACACTGTGTTTTTATTCAGCAAA

CNV 3

c.2160+530_2570del

short tandem repeat

proximal	TTATTTACTTATCATCATCATGGTCACCTCCTACATGGCTTCACCAAAAA
junction	TTATTTACTTATCATCATCATGGTCACCTCCTACATGGCTTCACCAAAAA
distal	ACGAATTCAGCTTCCTGCTGTCCATGCAGATGATGCTCCTTGATGCTGCT

short tandem repeat

proximal	ACATTCTGCTGCCTGAAAAAGCT**CC**AACACCTCTCTCTCTTTTAAAGGAT
junction	ACATTCTGCTGCCTGAAAAAGCT**CC**TTGATCAGGTGTTTCCAGGTAAGCA
distal	GTCTATGGCTTACTCGCTTGGTA**CC**TTGATCAGGTGTTTCCAGGTAAGCA

proximal	GGAATTTGGAGTCCATCCTTCCTCAGTGATAAGGAGTTTTTATAGCCACA
junction	TCCTCCTCTATAGGGTAAAGGTAATTGAGTTCTTCAGATCCCCAGCCCTC
distal	TCCTCCTCTATAGGGTAAAGGTAATTGAGTTCTTCAGATCCCCAGCCCTC

CNV 4

c.2918+532_3329-622delinsC

Oligo(G)n tract

proximal	TTTTTTGAGATAGTAACCATTACCCAT**GCTGGAG**G**GCAGTGG**T**GC**G**ATCT**
junction	TTTTTTGAGATAGTAACCATTACCCAT**GCTGGAG**G**GCAGTGG**T**GC**G**ATCT**
distal	GAGACAGAATCTCGCTCTGTCGCGCAG**GCTGGAG**T**GCAGTGG**C**GC**A**ATCT**

Alu repeat (AluSx)

proximal	TGG**CTCACTGCAA**C**CTCCGCCTCC**CA**GG**A**TC**A**AGCGATTCTCC**CA**CCTCA**
junction	TGG**CTCACTGCAA**C**CTCCGCCTCC**CG**GG**T**TC**G**AGCCATTCTCC**TG**CCTCA**
distal	CCA**CTCACTGCAA**G**CTCCGCCTCC**TG**GG**T**TC**G**AGCCATTCTCC**TG**CCTCA**

Alu repeat (AluY)

proximal	**GCCTCC**CA**AGTAGGTGGGACTACAGGTGC**ATA**CCACCA**TGCCC**AGCTAAT**
junction	**GCCTCC**TG**AGTAGCTGGGACTACAGGTGC**CCG**CCACCA**CGCCT**AGCTAAT**
distal	**GCCTCC**TG**AGTAGCTGGGACTACAGGTGC**CCG**CCACCA**CGCCT**AGCTAAT**

CNV 6 c.3863-553_4539+578del

proximal TTTTTGTTTGTTTGTTTGGTTTTCTTTGTGCTGTTTGAGAAGGAGTCTTG
junction TTTTTGTTTGTTTGTTTGGTTTTCTTTGTGCTGTTTGAGAAGGAGTCTTG
distal TGTTTGAATTTCTTTTTTTTTTTTTTTTTTTTTTTTTGAGACAGAGTCTCT

Alu repeat (AluSc8)
proximal CTCTGTCACCCAGGCTGGAGTGCAGTCTTGCAATCTCGGCTTACGCAACC
junction CTCTGTCACCCAGGCTGGAGTGCAGCGGTGTGATCTCGGCTCACTGCAAC
distal CTCTGTCACCCAGGTTGGAGTGCAGCGGTGTGATCTCGGCTCACTGCAAC
Alu repeat (AluSz6)

proximal TCCACCTCCTGGGTTCAAGTGATTCTCCTGCCTCAGCCTCCCGAGTAGTT
junction CTCTGCCTCCCAGGTTCAATTCTTGTGCCTCAGCCCCCCCGAGTAGTGGG
distal CTCTGCCTCCCAGGTTCAATTCTTGTGCCTCAGCCCCCCCGAGTAGTGGG

CNV 7 c.4254-197_4672delinsGCTTTTT

Oligo(G)n tract
proximal ACTGCTTGGGAAGGCCGAATGGGGAAAGGAATGCAAAGCTTAGGTGAATGGG
junction ACTGCTTGGGAAGGCCGAATGGGGAAAGGAATGCAAAGCTTAGGTGAATGGG
distal AACCCGAGAGAGCTACTAGTAGGCGTGAAGTTCGTGGCCCTGGTCTGAGGAT
Oligo(G)n tract

proximal TTGAAGCGCCATCTTTTTGAGGC------ATAGGTGACATGCCATCAGACCA
junction TTGAAGCGCCATCTTTTTGAGGCCTTTTTGAGGAATTTCCATTGGAGGAAAG
distal TTCCTGTTTCCTTGTCAGGTATG------GAGGAATTTCCATTGGAGGAAAG
Oligo(G)n tract

proximal CTGCGAGTGTTCAGGCAGCCTACCGCACTCCCAGGAGAGCTAGCGCCATCCC
junction CTCCCAGTCGTCCCCATCACGGGGGAAGCACTTGTTGGGTTTTTAAGCGACC
distal CTCCCAGTCGTCCCCATCACGGGGGAAGCACTTGTTGGGTTTTTAAGCGACC

CNV 8 c.4352+123_4540-585del

Oligo(G)n tract
proximal GACCCAACGGTGGGTTCAAGATGGCAGGCGTTGGGGAGGCCCCACTCAAT
junction GACCCAACGGTGGGTTCAAGATGGCAGGCGTTGGGGAGGCCCCACTCAAT
distal CAGGAACATTTCTCCGAGGAATAAAATTTGAAGCGAGCCATGAGAGGGTC

proximal CCTGCTCTGCTGGTCACTTCCATGTCTCTGACCAGCACTCCCCCAACCTC
junction CCTGCTCTGCTGGTCACTTCCATGTGAACAGCCAAGCACAAAGCTGCACC
distal TACAGGTAGAGTTCCCAGGCAGAGTGAACAGCCAAGCACAAAGCTGCACC
MIR2/LINE2

proximal TCCTTCCACACTTGTGTGCAGGGACATTCACTACCTCCTAGGAAGCCCCC
junction AGGAGAGAGAGGTGCTCGCCGAGAGACAGGGAGGGGAGTGTGGCAGGTGA
distal AGGAGAGAGAGGTGCTCGCCGAGAGACAGGGAGGGGAGTGTGGCAGGTGA
Oligo(G)n tract

CNV 9 c.4539+872_4635-565delins28

inverted repeat
proximal GCGTCAGCCACTGCTCCTGGCCTAAAGTTACTTTAAATTAACTGATCTCCCATTATTCG
junction GCGTCAGCCACTGCTCCTGGCCTAAAGTTACTTTAAATTAACTGATCTCCCATTATTCG
distal GAATAAATAGAATGCAATAAACAGCAACCATCCCAAACTGAGTTCTCAGCAGCAAATCT

L1ME4A
proximal CCACTTAGGTTTTTT----------------------------AGTTTTCACCATTATA
junction CCACTTAGGTTTTTTGTGCACCTTGTTTCTCTCTCTCCTGCCAGGATTTTGTGCGTGTG
distal CCAGTATGAAATTTT----------------------------GGATTTTGTGCGTGTG
Z-DNA motif

proximal AGCAATGCTATGATGTACATTCAAATGGAAATGTGTTTACACACTTATTAACAGTCTTA
junction TGCTTAAAGGTGGATGACAATGACAGTTCATGGGATTGAGCTCTGGGGTCCAGAGTTGG
distal TGCTTAAAGGTGGATGACAATGACAGTTCATGGGATTGAGCTCTGGGGTCCAGAGTTGG
Oligo(G)n tract
inverted repeat

CNV 10 c.4540-1000_4635-389delinsTGCCCG

proximal TTGACAGAGTGAATGCTTTTATGAGAATGTAAGCGGAATGTGTTCCCAGATT
junction TTGACAGAGTGAATGCTTTTATGAGAATGTAAGCGGAATGTGTTCCCAGATT
distal TTACCCTTGATTGACTGAATGTCAGTGCCTTAACTTTGGGCTGTGGAGTGAG

proximal TGCAGTAATATTGCCACCTGGTG------**G**ACAAACCCATGCACCTTTGAAT
junction TGCAGTAATATTGCCACCTGGTG**TGCCCGG**GTGGTTGTTAGAGTCTCATTTT
distal TCGGAACTCCCCCGAGGTGTGCA------**G**GTGGTTGTTAGAGTCTCATTTT
Oligo(G)n tract

proximal TTTCCAAAATATTTCGATGAACTAGCTTCCAGTCCTAGATGTATTTTGAAAG
junction TGCAGGGTGGAAGACAGGAGGGCTGCAGCCTTCATTCCACACTGACATGGTC
distal TGCAGGGTGGAAGACAGGAGGGCTGCAGCCTTCATTCCACACTGACATGGTC
Oligo(G)n tract

CNV 11 c.5864_6085del

proximal CTCTCACCCTGTCTCAGTTCTCAGTCCGGTTTCTTCGTATCTTGCAGATT
junction CTCTCACCCTGTCTCAGTTCTCAGTCCGGTTTCTTCGTATCTTGCAGATT
distal TAACCAATATTTCTGAAGTCCATCAAAATATGGGCTACTGTCCTCAGTTT

Oligo(G)n tract
proximal TATCCAGGCACCTCCAGCCCAG**CAG**TGGACAGGCTGTGTGTCGGAGTTCG
junction TATCCAGGCACCTCCAGCCCAG**CAG**GACGAGAACATCTTTACCTTTATGC
distal GATGCAATTGATGAGCTGCTCA**CAG**GACGAGAACATCTTTACCTTTATGC

proximal CCCTGGAGAGGTGGGTACTCTGCAGACCACGTGTGAAAGGCTTCCGAACA
junction CCGGCTTCGAGGTGTACCAGCAGAAGAAATCGAAAAGGTGAAAAATGTTT
distal CCGGCTTCGAGGTGTACCAGCAGAAGAAATCGAAAAGGTGAAAAATGTTT

CNV 12 c.66+520_67-389dup

proximal GCACATTGTTTTTCACATGTTAGCATTTCTGAAATTGGGATGCAGCTCA
junction GTCACCTGCCTCCTCTGAGCCTCTTTCCTTAACTGCAGAGTGAGTGGCTA
distal GTCACCTGCCTCCTCTGAGCCTCTTTCCTTAACTGCAGAGTGAGTGGCTA

MER104
proximal CGATCAAGTCACAGTTTAACTGGAC**A**CATTATTTTTCTTTCTTAGTGGTG
junction CAGAGAAATCTTTACTACCTGTTAG**A**CATTATTTTTCTTTCTTAGTGGTG
distal CAGAGAAATCTTTACTACCTGTTAG**A**TCAGCATTACCTGGGAGCTTGTTA
MER5A1

proximal CAGAAAAGTAACAGTGTGTCTTACAATTGACTGCGTCCTAGATTCTGTGA
junction CAGAAAAGTAACAGTGTGTCTTACAATTGACTGCGTCCTAGATTCTGTGA
distal GAAATGCAAGCTCTGGTGGGGCCATACTGAACCCAAATCTGCATTCATGT

CNV 13 c.768+6839_858+66dup

proximal TTGATGAACACATTTGATCCAAAGCATCAGAAATACTATGCCAACAAGAC
junction TTCAAGAGGTGAGTAGCCTGATGGTAGTAATTATAATTTCATTATGTCTT
distal TTCAAGAGGTGAGTAGCCTGATGGTAGTAATTATAATTTCATTATGTCTT

proximal TTTTTAGGAGGTGATAAACA**TGTC**TGTTCTACCTTAAGAAAAAAAATATTA
junction TCCACCATTTACCCCACTTA**TGTC**TGTTCTACCTTAAGAAAAAAAATATTA
distal TCCACCATTTACCCCACTTA**TGTC**AAATAATTTAATTGTATTTCAAACCT

proximal CACAGTCCCAAGGGAGAGACATGGTTTTGATCCCAGACAACCCAAGCAGA
junction CACAGTCCCAAGGGAGAGACATGGTTTTGATCCCAGACAACCCAAGCAGA
distal GTTCAAGGAAAAGTACATTTGATCTTTCCATCTAGCAATTTCAAAGCACC

CNV 14 c.6006-29_6370dup

proximal AAGCAGCTTCCTTTTCCCGTTGGCAGAAGCTTCTCCAGCCCTAGCTCTAT
junction CAGGGATGGACCCCCAGGCACGCCGCATGCTGTGGAACGTCATCGTGAGC
distal CAGGGATGGACCCCCAGGCACGCCGCATGCTGTGGAACGTCATCGTGAGC

proximal GGTCATCCCTCCACTCCTTGAAG**G**ATACTCAGTAATTGCTTTTTTTCTTG
junction ATCATCAGAGAAGGGAGGGCTGT**G**ATACTCAGTAATTGCTTTTTTTCTTG
distal ATCATCAGAGAAGGGAGGGCTGT**G**GTCCTCACATCCCACAGGCAAGAGAT
Oligo(G)n tract

proximal CAGTATTTTAACCAATATTTCTGAAGTCCATCAAAATATGGGCTACTGTC
junction CAGTATTTTAACCAATATTTCTGAAGTCCATCAAAATATGGGCTACTGTC
distal TCCCAGGGCTGGGGAAGGTGGGTGGGAATCCTCTCCTGCTCACCTCCTCT
Oligo(G)n tract

CNV 15 c.6147+411_c.6479+293delins4583_5715-778

5’ breakpoint (intron 44 - intron 31)

proximal TGTAATTGCAAAATGCTGGAAGTTACCTAAATGTTTAGTCATTGTAGATT
junction TGTAATTGCAAAATGCTGGAAGTTACCTAAATGTTTAGTCATTGTAGATT
distal AGGTAATTAACGTGGGTGTCTCATTGCCTCAGAGAACACAGCGCAGCACG

LINE1
proximal GGCTGAATAATTTATGGT**ACAGACA**CACAATAAAGTCTTACGCAACTATA
junction GGCTGAATAATTTATGGT**ACAGACA**GGAACATCTCCGACTTCTTGGTAAA
distal GAAATTCTACAAGACCTG**ACGGACA**GGAACATCTCCGACTTCTTGGTAAA

short tandem repeat
proximal AAAAAGAAGAAGAAAAGTCTCAGTAAACTGATATGGAGATATTTCCAGTA
junction AACGTATCCTGCTCTTATAAGAAGCAGGTAAGAAGAAATCCTTTTATGCT
distal AACGTATCCTGCTCTTATAAGAAGCAGGTAAGAAGAAATCCTTTTATGCT

3’ breakpoint (intron 40 - intron 47)

Oligo(G)n tract
proximal CCTAAAGAGGACCAGAGAAGTACACCATAGTCCCTGGCTTCCAGAAGGTC
junction CCTAAAGAGGACCAGAGAAGTACACCATAGTCCCTGGCTTCCAGAAGGTC
distal CCAAGTCTCTGGATGGGGCATCTGATCAGGATGCATGCAGAGCCTGGCTG
Oligo(G)n tract

LINE2 mirror repeat Oligo(G)n tract
proximal AGGGAGGGTTTCAAAGAAGAGG**CTG**TGTCTTTAAGAATGGGGAAGATTCC
junction AGGGAGGGTTTCAAAGAAGAGG**CTG**CCTCAATATTTCACCACTTATCTCA
distal GGATGAGGGAGGGCTGCTACCA**CTG**CCTCAATATTTCACCACTTATCTCA
Oligo(G)n tract

Oligo(G)n tract
proximal ATTTGGTGGGGCAGGAGGAGGAGAACATTGAGGGACTGGAAACACATGCG
junction ACAGATCCGGGACCTGTGGCCTATTTACTAAGAGTCCACTCCAATGTAGG
distal ACAGATCCGGGACCTGTGGCCTATTTACTAAGAGTCCACTCCAATGTAGG

Figure S15: Breakpoint analysis of CNVs identified and validated in the study.
The presence of microhomology at the breakpoints was assessed using ClustalW to align the junction fragment (central line) and the proximal (in cyan) and distal (in grey) breakpoint regions (150 bp). Microhomology regions are reported in bold red text, while inserted nucleotides at the juctions are in dark blue. DNA motifs and repetitive elements identified by bioinformatic analysis are graphically represented in different colors. Repetitive elements, such as *Alu* repeats and LINEs, are represented in light green, oligo(G) tracts in purple and repeat motifs in orange.

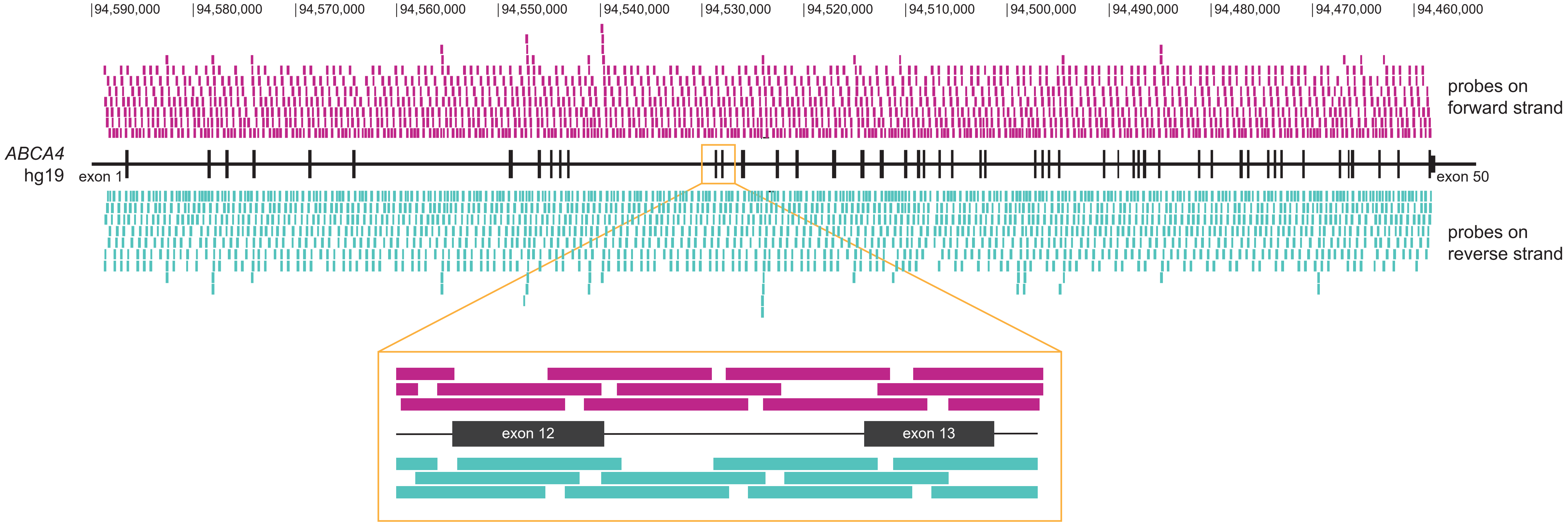

Figure S16: Schematic representation of the smMIPs panel covering *ABCA4*. The yellow box reports a zoom in of the region encompassing exon 12 to exon 13, showing that each nucleotide position is targeted (on average) by five probes.
