# Supplementary material for: Novel and Recurrent Copy Number Variants in ABCA4-Associated Retinopathy
Source: Int J Mol Sci. 2024 May 29;25(11):5940. doi: 10.3390/ijms25115940 (PMC11173210; doi:10.3390/ijms25115940)
Supplement: Supplementary file 1 [file ijms-25-05940-s001.zip › Tables.pdf]

Table S1: Details on previous screening methods and known variants in patients carrying a newly identified copy number variants (CNV).

					Additional pathogenic variants	
**Patient ID**	**Sample origin**	**Previous screening**	**CNV ID**	**CNV identified in this study**	**cDNA notation**	**Protein notation**
**067513**	Basel, Switzerland	smMIPs_1	CNV 9	c.4540-1000_4635-389delinsTGCCCG	c.2894A>G	p.(Asn965Ser)
**068592**	Ghent, Belgium	smMIPs_1	CNV 4a	c.(2918+757_2918+981)_(3329-420_3329-644)del	c.[5461-10T>C;5603A>T]	p.[[Thr1821Aspfs*6,Thr1821Valfs*13];(Asn1868Ile)]
**073768**	Ljubljana, Slovenia	smMIPs_2	CNV 12	c.66+520_67-389dup	c.5714+5G>A	p.[=,Glu1863Leufs*33]
**075728**	Naples, Italy	smMIPs_1, Clinical exome	CNV 12	c.66+520_67-389dup	c.247_250dup	p.(Ser84Thrfs*16)
**079908**	Lille, France	dHPLC	CNV 2	c.1239+303_1555-5571del	c.656G>C;[2588G>C;5603A>T]	p.(Arg219Thr);[Gly863Ala,Gly863del;(Asn1868Ile)]
**079919**	Lille, France	NGS_1	CNV 1	c.699_768+341del	c.2942C>T	p.(Pro981Leu)
**079920**	Lille, France	NGS_2	CNV 14	c.6006-29_6370dup	c.5882G>A	p.(Gly1961Glu)
**079921**	Lille, France	NGS_3	CNV 1	c.699_768+341del	c.[5560G>T;5882G>A]	p.[(Val1854Leu);(Gly1961Glu)]
**079922**	Lille, France	NGS_4	CNV 11	c.5864_6085del	c.3323G>A	p.(Arg1108His)
**079923**	Lille, France	NGS_4	CNV 4b	c.2918+532_3329-622del	c.5882G>A	p.(Gly1961Glu)
**079924**	Lille, France	NGS_4	CNV 7	c.4254-197_4672delinsGCTTTTT	c.710T>C	p.(Leu237Pro)
**079925**	Lille, France	NGS_5	CNV 10	c.4539+872_4635-565delins28	c.[3322C>T;6320G>A]	p.[(Arg1108Cys);(Arg2107His)]
**079926**	Lille, France	NGS_5	CNV 13	c.768+6839_858+66dup	c.4919G>A	p.(Arg1640Gln)
**079927**	Lille, France	NGS_5	CNV 8	c.4352+123_4540-585del	c.3386G>T	p.(Arg1129Leu)
**079928**	Lille, France	NGS_5	CNV 5	g.(?_94458389)_(94505684_94506764)del	c.71G>A	p.(Arg24His)
**079929**	Lille, France	NGS_5	CNV 3	c.2160+531_2569del	c.5882G>A	p.(Gly1961Glu)
**079930**	Lille, France	NGS_6	CNV 15	c.6147+411_c.6479+293delins4583_5715-778 (*)	c.5817G>T (*)	p.(Arg1939Ser)
**079932**	Lille, France	NGS_6	CNV 6	c.3863-553_4539+578del	c.5882G>A	p.(Gly1961Glu)
**081420**	Naples, Italy	WES	CNV 12	c.66+520_67-389dup	c.6088C>T	p.(Arg2030*)

Legend: smMIPs_1: RP-LCA smMIPs panel (Panneman et al, 2023; PMID: 36819107); smMIPs_2: MD smMIPs panel (Hitti-Malin et al, 2022; PMID: 36259723); dHPLC: dHPLC 17 exons of ABCA4 and Sanger 33 exons; NGS_1: Next generation sequencing of *ABCA4, PRPH2, ELOVL4, BEST1, RHO* and *RS1*; NGS_2: Next Generation Sequencing by Ion Torrent of PGM*, ABCA4, PRPH2, ELOVL4, BEST1, RHO* and *RS1*; NGS_3: Next generation sequencing of 156 inherited retinal disease genes; NGS_4: Next generation sequencing of 18 inherited retinal disease genes; NGS_5: Next generation sequencing (Haloplex) of 22 genes and 33 *ABCA4* intronic variants; NGS_6: Next generation sequencing (Haloplex) of 230 genes and 79 intronic variants *in ABCA4, CEP290, USH2A* and *RP17*; WES: whole exome sequencing. (*) variant in homozygosity.

Table S2. Overview of known structural variants (SV) in *ABCA4.* Novel copy number variants (CNVs) identified in this study are reported in bold. In column 5, '# reports', the numbers in brackets refer to the newly identified alleles in this study.

Region	cDNA notation	Genomic DNA (hg19) notation	Type of SV	# reports	Reference (PMID)
w.g.	n/a	g.(?_94458792)_(94586602_?)del	del	1	28044389, 16917483
w.g.	n/a	g.93811483_98850435del	del	1	25533962
?_int1	n/a	g.(94578623_94586535)_(94586602_?)del	del	1	32307445
int1	c.66+520_67-389dup	g.94579013_94586018dup	dup	1 (3)	30670881, **This study**
int1_int2	c.67-1845_160+345del	g.94578188_94580471del	del	1	28446513
int1_int2	c.67-140_161-355del	g.94577492_94578764del	del	2	35657619 , 37774808
int1_int6	c.67-975_769-4582dup;769-4582_769-4581insA	g.94553579_94579598dup;94553579_94553578insA	dup/ins	1	30670881
int4_int5	c.(442+1_443-1)_(570+1_571-1)del	g.(94564548_94568570)_(94568699_94574132)del	del	6	25363634, 25363634, 28044389, 31522899, 25444351
int4_int5	c.442+799_570+541del	g.94568035_94573339del	del	1	30670881
int4_int5	c.442+1194_571-1399del	g.94565946_94572939del	del	1	37705246
int4_int6	c.443-1219_768+1439del	g.94562910_94569916del	del	1	32307445
int5_int6	c.571-801_768+3062del	g.94561288_94565348del	del	1	32307445
ex6_int6	c.699_768+341del	g.94564009_94564419del	del	12 (2)	37779911, 37705246, 32307445, 32619608, **This study**
ex6_int6	c.742_768+29del	g.94564321_94564376del	del	2	28044389, 23755871
int6_int7	c.768+6839_858+66dup	g.94548847_94557516dup	dup	1 (1)	37705246, **This study**
int6_int7	c.(768+1_769-1)_(858+1_859-1)del	g.(94546275_94548907)_(94548998_94564349)del	del	5	22247458, 26377081, 29925512, 24550365
int7_ex8	c.859-45_952delinsTCTGACC	g.94546181_94546319delinsGGTCAGA	del	4	28044389, 12202497
int9_int11	c.1239+291_1555-5574del	g.94534447_94544587del	del	1	30670881
int9_int11	c.1239+303_1555-5571del	g.94534444_94544575del	del	1	**This study**
int10_int16	c.(1356+1_1357-1)_(2587+1_2588-1)del	g.(94517255_94520666)_(94543444_94544145)del	del	2	28044389, 10874631
int11_int12	c.(1356+1_1357-1)_(1554+1_1555-1)del	g.(94528874_94543245)_(94543444_94543245)del	del	1	32619608
int11_int13	c.(1554+1_1555-1)_(1937+1_1938-1)del	g.(94526316_94528132)_(94528874_94543245)del	del	3	29555955, 30902645, 33214501, 32646556
int11_int13	c.1555-3491_1938-83delins1734_1761-107inv	g.94526398_94532364delins94528694_94528416inv	del/inv	1	32307445
int11_int13	c.1555-1033_1937+615delinsAGC	g.94527518_94529906delinsGCT	del	1	32307445
int11_int14	c.1555-2428_2161-101delins2160+7_2160+230invATGAATGins	g.94522479_94531301delins94525863_94526086invCATTCATins	del/inv	1	32307446
int12_int13	c.(1760+1_1761-1)_(1937+1_1938-1)del	g.(94526316_94528132)_(94528310_94528667)del	del	5	29555955, 30902645, 33214501, 26720470
**int14_int16**	**c.2160+531_2569del**	**g.94520685_94525562del**	**del**	**1**	**This study**
int14_int17	c.(2160+1_2161-1)_(2653+1_2654-1)del	g.(94514514_94517188)_(94522379_94526092)	del	1	32893963
int15_int16	c.(2382+1_2383-1)_(2587+1_2588-1)del	g.(94517255_94520666)_(94520872_94522156)del	del	2	32307445, 32036094
int16_ex17	c.2588-161_2615del	g.94517231_94517419del	del	1	32531858
int17_int18	c.2654-905_2743+35del	g.94514390_94515419del	del	4	28044389, 10746567, 12754711
int17_int19	c.(2653+1_2654-1)_(2918+1_2919-1)del	g.(94510301_94512474)_(94514514_94517188)del	del	1	31799409, 33185728
int17_?	n/a	g.(?_94458792)_(94514514_94517188)del	del	2	32307445, 31766579
**int19_int22**	**c.2918+532_3329-622del**	**g.94507580_94511943del**	**del**	**1**	**This study**
int19_int22	c.2918+552_3329-601del	g.94507560_94511924del	del	1	37705246
int19_int22	c.(2918+765_2918+775)_(3328+618_3328+662)del	g.(94507655_94507699)_(94511700_94511710)del	del	2	33546218
int19_int22	c.2918+775_3328+640del	g.94507682_94511705del	del	1	30670881

Table S2. continued

Region	cDNA notation	DNA (hg19) notation	Type of SV	# reports	Reference (PMID)
**int19_int22**	**c.(2918+757_2918+981)_(3329-644_3329-420)del**	**g.(94507378_94507602)_(94511494_94511718)del**	**del**	**1**	**This study**
int19_int22	c.(2918+1_2919-1)_(3328+1_3329-1)del	g.(94506959_94508316)_(94510301_94512474)del	del	25	25363634, 30903310, 28044389, 29555955, 29971439, 26720470, 25444351, 28002570, 28355279, 31522899, 32646556
ex20_int22	c.2999_3328+610del	g.94507707_94510220del	del	2	28559085
ex20_ex23	c.3033_3364del	g.94506923_94510186del	del	1	32307445
int23_?	n/a	g.94402743_94505682del	del	1 (1)	28041643, **This study**
**int26_int30**	**c.3863-553_4539+578**	**g.94494423_94498152del**	**del**	**1**	**This study**
int27_int30	c.(4128+1_4129-1)_(4539+1_4540-1)del	g.(94490605_94495000)_(94496677_94497333)del	del	1	32307445
int27_int43	c.4128+246_6006-716dup	g.94471859_94497093dup	dup	1	35657619
int28_ex33	c.4254-197_4672delinsGCTTTTT	g.94487503_94496279delinsAAAAAGC	del	1 (1)	32307445, **This study**
**int29_int30**	**c.4352+123_4540-585del**	**g.94491189_94495861del**	**del**	**1**	**This study**
int29_int35	c.(4352+1_4353–1)_(5018+1_5019-1)dup	g.(94485316_94486795)_(94495188_94495983)dup	dup	1	32307445
ex30_ex38	c.4353_5460del	g.94480099_94495187del	del	1	32307445
**int30_int31**	**c.4539+872_4635-565delins28**	**g.94489539_94494129delins28**	**del**	**1**	**This study**
**int30_int31**	**c.4540-1000_4635-389delinsTGCCCG**	**g.94489363_94491604delisCGGGCA**	**del**	**1**	**This study**
int31_int40/ int44_int47	c.[(4634+1_4635-1)_(5714+1_5715-1)dup;(6147+1_6148-1)_(6479+1_6480-1)del]	g.(94474428_94490509)_(94488975_94490509)dup;(94463667_94466391)_(94467549_94470996)del	delins	5	29736279
**ex31_int40/ int44_int47**	**c.6147+411_c.6479+293delins4583_5715-778**	**g.94466098_94470586delins94475205_94490561**	**delins**	**1**	**This study**
int32_int34	c.4667+507_4849-94del	g.94487059_94488435del	del	1	37705246
int36_int37	c.5196+1483_5313-89del	g.94480338_94483658del	del	1	32646556
int37_int44	c.5313-85_6147+21dup	g.94470977_94480332dup	dup	1	35657619
int37_int44	c.5313-1_6147+1dup	g.94470996_94480247dup	dup	1	33090715
int39_int41	c.(5584+1_5585-1)_(5835+1_5836-1)dup	g.(94473854_94474306)_(94476486_94476817)dup	dup	1	33090715
int39_?	n/a	g.94457537_94476649del	del	1	30670881
int38_int49	c.(5460+1_5461-1)_(6816+1_6817-1)del	g.(94458799_94461664)_(94476942_94480098)del	del	1	32307445
int39_?	n/a	g.(?_94458792)_(94476486_94476817)del	del	1	32307445
int40_int41	c.(5714+1_5715-1)_(5835+1_5836-1)del	g.(94473854_94474306)_(94474428_94476355)	del	1	36259723
**ex42_ex44**	**c.5864_6085del**	**g.94471059_94473825del**	**del**	**1**	**This study**
int43_int45	c.(6005+1_6006–1)_(6282+1_6283–1)dup	g.(94466662_94467413)_(94471139_94473189)dup	dup	1	32307445
int43_int44	c.6005+658_6147+757delinsTTTAACAGTGTT	g.94470240_94472532delinsAACACTGTTAAA	del	1	32307445
**int43_ex46**	**c.6006-29_6370dup**	**g.94466574_94471167dup**	**dup**	**1**	**This study**
int44_int47	c.(6147+1_6148-1)_(6479+1_6480-1)del	g.(94463667_94466391)_(94467549_94470996)del	del	1	23484092
int44_ex48	c.6148-698_6670delinsTGTGCACCTCCCTAG	g.94463476_94468246delinsCTAGGGAGGTGCACA	del	5	29526278, 28044389, 26527198
int45_ex48	c.6282+63_6546del	g.94467351-94463600	del	1	32307445
int48_?	n/a	g.(?_94458792)_(94461752_94463416)del	del	1	32307445
int49_?	n/a	g.(?_94458792)_(94458799_94461664)del	del	1	30718709

Legend: # = number of, del = deletion, dup = duplication, ex = exon, ins = insertion, int = intron, inv = inversion, n/a = not applicable, w.g. = whole gene.

Table S3: Breakpoint analysis of identified CNVs, including microhomology and DNA motifs and repetitive elements at the proximal and distal breakpoints. CNV 4a and CNV 5 were excluded from the analysis as no precise breakpoints were identified. *Alu/Alu* identity was assessed by comparing the consensus sequences of the Alu repeats, obtained through Dfam, using BLAST2.

CNV ID	Variant (cDNA)	Microhomology	Insertion	Breakpoint	Repetitive elements	Oligo(G)n tract	Other non-B DNA motifs (#)	Potential underlying mechanisms
**CNV 1**	c.699_768+341del	2 nt	--	Proximal	--	1 (spanning the b.p.)	--	Replicative/NHEJ
				Distal	MIRb (SINE)	1	--	
**CNV 2**	c.1239+302_1555-5570del	3 nt	--	Proximal	--	2	--	Replicative/NHEJ
				Distal	--	1	Inverted repeat (1)	
**CNV 3**	c.2160+530_2570del	2 nt	--	Proximal	--	--	Short tandem repeat (2)	Replicative/NHEJ
				Distal	--	--	--	
**CNV 4b**	c.2918+533_3329-622del	*Alu/Alu*	--	Proximal	AluSx (Alu repeat)	1	--	NAHR/Replicative
				Distal	AluY (Alu repeat)	--	--	
**CNV 6**	c.3863-553_4539+578del	*Alu/Alu*	--	Proximal	AluSc8 (Alu repeat)	--	--	NAHR/Replicative
				Distal	AluSz6 (Alu repeat)	--	--	
**CNV 7**	c.4254-197_4672delinsGCTTTTT	--	7 nt	Proximal	--	1	--	NHEJ
				Distal	--	2 (1 spanning the b.p.)	--	
**CNV 8**	c.4352+123_4540-585del	2 nt	--	Proximal	--	1	--	Replicative/NHEJ
				Distal	MIR2 (LINE)	1	--	
**CNV 9**	c.4539+872_4635-565delins28	3 nt	28 nt	Proximal	L1ME4A (LINE)	--	Z-DNA motif (1)	Replicative/NHEJ
				Distal	--	1	Inverted repeat (1)	
**CNV 10**	c.4540-1000_4635-389delinsTGCCCG	1 nt	6 nt	Proximal	--	--	--	Replicative/NHEJ
				Distal	--	2 (1 spanning the b.p.)	--	
**CNV 11**	c.5864_6085del	3 nt	--	Proximal	--	1	--	Replicative/NHEJ
				Distal	--	--	--	

**CNV 12**	c.66+520_67-389dup	1 nt	--	Proximal	MER104 (DNA transposon)	--	--	Replicative/NHEJ
				Distal	MER5A1 (DNA transposon)	--	--	
**CNV 13**	c.768+6839_858+66dup	4 nt	--	Proximal	--	--	--	Replicative/NHEJ
				Distal	--	--	--	
**CNV 14**	c.6006-29_6370dup	1 nt	--	Proximal	--	--	--	Replicative/NHEJ
				Distal	--	2	--	
**CNV 15_5'bp**	c.6147+411_c.6479+293 delins4583_ 5715-778	7 nt	--	Proximal (int44)	L1ME4A (LINE)	--	Short tandem repeat (1)	Replicative/NHEJ
				Distal (ex31)	--	--	--	
**CNV 15_3'bp**		3 nt	--	Proximal (int40)	L2 (LINE)	3	Mirror repeat (1) spanning the b.p.	
				Distal (int47)	--	2	--	

Legend: (#) = number identified, b.p. = breakpoint, SINE = short interspersed element, LINE = long interspersed element, NAHR = Non-allelic homologous recombination, NHEJ = includes non-homologous end joining (NHEJ) but also microhomology-mediated end joining (MMEJ), Replicative = Replication-based mechanisms such as fork stalling and template switching (FoSTeS), microhomology-mediate break-induced replication (MMBIR), serial replication slippage (SRS) and break-induced SRS (BISRS).

Table S4. Oligonucleotides employed in this study. **A.** Primers for breakpoint confirmation of identified deletions. **B.** Primers for breakpoint confirmation of identified duplications and a complex rearrangement.

A.

CNV breakpoints	Genomic position	Sequence(5'-3')
CNV 1	Intron 5_Fwd	GCTACTTCTCAACCCCACCT
	Intron 6_Rev	AGGATAGATTCTGGGCCTGG
CNV 2	Exon 9_Fwd	TTTGCTGATGGGAAAAATCC
	Intron 11_Rev	ATCCCGAATGTCATCCTCCC
CNV 3	Intron 14_Fwd	CCTTTCCCCTTTCCTCCTGT
	Intron 16_Rev	TGTGGGGAGAGAAATGTGCT
CNV 4	Intron 19_Fwd	ATCAAGCGATTCTCCCACCT
	Intron 22_Rev	GGGTTGGGCATTGTTGGTAA
CNV 6	Intron 26_Fwd	GGGAACTGAGCTCACAGAGG
	Intron 30_Rev	CCTGTTTGGGAACAGTTTATTC
CNV 7	Intron 27_Fwd	AGATTCTTCAGTGGCCACCA
	Intron 34_Rev	TCTGAGACGCTGCACTAACA
CNV 8	Intron 28_Fwd	TGGGTCACATGCTGCTTAGA
	Intron 31_Rev	AATGGGGCCCTCAAATCAGA
CNV 9	Intron 30_Fwd	GTTTGCAGCCACCCAAAC
	Intron 32_Rev	CCCACACTCCCTGAAGCTTA
CNV 10	Intron 30_Fwd	GTGTCCGTGGTTGATAATGG
	Intron 32_Rev	GTGTCTTCTGAGTCTGGGATGT
CNV 11	Intron 40_Fwd	CTCTGGACATGAGCACCTGA
	Intron 44_Rev	GCAAAGGTAAGCAGACATACGT

B.

CNV breakpoints	Genomic position	Sequence(5'-3')
CNV 12	Intron 1_Fwd	TTTCTGAAATTGGGATGCAG
	Intron 1_Rev	GTTTTCCCAGGCAGAACAGA
	Intron 1_Fwd	TCAGCAGCCTCTTCCAATGT
	Intron 2_Rev	GGCCCAGACCAAAGTCTC
CNV 13	Intron 6_Fwd	AGACACATTGAACCTGAGGCT
	Intron 6_Rev	ACACACACACACACAATTTCCT
	Intron 7_Fwd	ACACTCCTAGACAGCCGTTC
	Intron 7_Rev	TTTGGGATGTGAACAGGTGC
CNV 14	Intron 43_Fwd	ACCCTACTTGCTTCCCTGAG
	Exon 44_Rev	TGTTCTCGTCCTGTGAGCAG
	Exon 45_Fwd	GACTGTCTACGCCGACTGC
	Intron 47_Rev	CTCTTCCAAGTGTCAATGGAG
CNV 15	Intron 31_Fwd	TTTTGGAAGACAACAAGCAG
	Intron 32_Rev	GTGTCTTCTGAGTCTGGGATGT
	Intron 40_Fwd	CAATGGTTACAGGGTATCCAAA
	Intron 40_Rev	CTCCTGAGGAAAGAAATGACC
	Exon 44_Fwd	GGGCTACTGTCCTCAGTTTG
	Intron 47_Rev	ATGCAGAGGAGAAAGGCTGT
